# Ankle and Foot Arthroplasty and Prosthesis: A Review on the Current and Upcoming State of Designs and Manufacturing

**DOI:** 10.3390/mi14112081

**Published:** 2023-11-10

**Authors:** Richa Gupta, Kyra Grove, Alice Wei, Jennifer Lee, Adil Akkouch

**Affiliations:** 1 Western Michigan University Homer Stryker M.D. School of Medicine, Kalamazoo, MI 49008, USA; richa.gupta@wmed.edu (R.G.); kyra.grove@wmed.edu (K.G.); alice.wei@wmed.edu (A.W.); jennifer.lee@wmed.edu (J.L.); 2 Department of Orthopaedic Surgery and Medical Engineering Program, Western Michigan University Homer Stryker M.D. School of Medicine, Kalamazoo, MI 49008, USA

**Keywords:** additive manufacturing, 3D printing, total ankle arthroplasty, total talus arthroplasty, ankle prosthesis, powered exoskeleton

## Abstract

The foot and ankle serve vital roles in weight bearing, balance, and flexibility but are susceptible to many diverse ailments, making treatment difficult. More commonly, Total Ankle Arthroplasty (TAA) and Total Talus Replacement (TTR) are used for patients with ankle degeneration and avascular necrosis of the talus, respectively. Ankle prosthesis and orthosis are also indicated for use with lower limb extremity amputations or locomotor disability, leading to the development of powered exoskeletons. However, patient outcomes remain suboptimal, commonly due to the misfitting of implants to the patient-specific anatomy. Additive manufacturing (AM) is being used to create customized, patient-specific implants and porous implant cages that provide structural support while allowing for increased bony ingrowth and to develop customized, lightweight exoskeletons with multifunctional actuators. AM implants and devices have shown success in preserving stability and mobility of the joint and achieving fast recovery, as well as significant improvements in gait rehabilitation, gait assistance, and strength for patients. This review of the literature highlights various devices and technologies currently used for foot and ankle prosthesis and orthosis with deep insight into improvements from historical technologies, manufacturing methods, and future developments in the biomedical space.

## 1. Introduction

The foot and ankle form a complex system of 28 bones and 33 joints and are involved in a wide variety of vital functions, including weight bearing, balance, shock absorption, and flexibility to ground terrain. The foot and ankle are common sites of emergent injury; for instance, ankle fractures account for over 20% of visits to the emergency room due to lower extremity fractures [1]. Injuries and ailments that affect the foot and ankle have been attributed to a wide variety of causes, ranging from high-energy trauma to end-stage osteoarthritis, making treatment of the injury difficult. Damage to the foot and ankle can be debilitating, causing pain and loss of function in bearing weight and walking for the patient. In recent years, it has been reported that the direct cost of managing ankle/foot fracture can reach up to almost USD 22,000/patient, while treatment of soft tissue injury averages around USD 2700/patient [2]. Long-term recovery prognosis is also reported as variable and suboptimal in the literature [3].

Prosthetic components have become increasingly popular, ranging from single bone/joint components to functional limb prosthetics. Accordingly, attention in the scientific community has been paid to improving foot and ankle prosthetic options. With discomfort due to improper fit being the most common cause of rejection of prosthetic devices [4], it is vital to produce dependable prosthetic devices for patients [5]. Additive manufacturing (AM), or the industrial process for three-dimensional (3D) printing, has become increasingly prevalent in the development of custom prosthetic devices for individual patients, thereby improving patient satisfaction.

A previously published review on the use of polymer-based AM [6] found that compared to traditional methods, AM methods had decreased costs, improved design and fabrication efficiency, and had better or similar functionality and patient satisfaction. AM prosthetics are typically lighter in weight than traditional prosthetics [7], which can be of benefit to patients with limited mobility. Furthermore, traditional manufacturing results in excess material and unnecessarily increased costs, and it produces inconsistent results [8]. This is due to a more time-consuming process involving taking anthropometric measurements, creating molds, constructing the prosthetic device using thermoplastic materials, and subsequently, making further adjustments to the device post-construction to improve comfort and functionality. Instead, with AM, optimal customization is possible using patient data scanning technology and 3D printing techniques [9]. Still, the structural and material integrity of AM prosthetics remain the main concerns of physicians [6].

**Figure 1:**
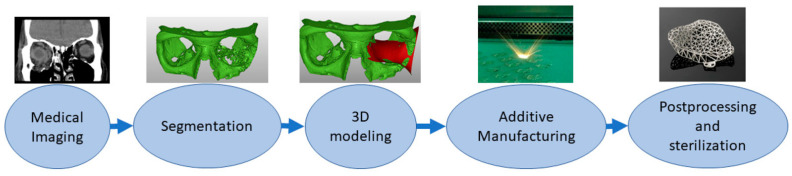
Schematic of the process of Additive Manufacturing of medical implants, outlining the process of acquisition of medical imaging data, creation and processing of a digital model, and creation of a 3D-printed physical product [11].

AM of implants is a multistep process [10,11], outlined in Figure 1. The first step entails medical image acquisition, commonly obtained using computerized tomography (CT) or magnetic resonance imaging (MRI) of the patient [10,12]. Next, a digital 3D reconstruction is generated and undergoes processing. During this stage, the internal and external geometries of the model can be modified, allowing for the inclusion of porous surfaces and for significant control over the weight and mechanical properties of the implant [10]. The model is then 3D printed into a physical product. There are several methods of 3D printing available for use, of which the most common include powder bed fusion (PBF), stereolithography (SLA), and fused deposition modeling (FDM).

PBF methods include Selective Laser Sintering (SLS), Direct Metal Laser Sintering (DMLS), Selective Laser Melting (SLM), and Electron Beam Melting (EBM), all of which utilize a focused laser or electron beam to fuse deposited particles layer-by-layer [13,14,15]. SLS uses polymer and ceramic powders, while DMLS, SLM, and EBM use metal/alloy materials. SLA utilizes the layering of a liquid-base resin that is sequentially cured, and FDM uses the extrusion of melted thermoplastic beads. SLS and EBM methods do not require the use of supports during manufacturing and are, therefore, recognized for their ability to construct complex geometries, beneficial in addressing patient-specific anatomy and pathologies. Furthermore, metals are more commonly utilized in load-bearing applications such as within the foot and ankle compared to polymer materials [10,16]. The advantage of using PBF technologies such as SLM is that they can result in lower wear-resistant titanium-based scaffolds when compared to specimens fabricated by EBM or casting methods [17].

The objective of this review is to outline the historical development and current state of treatments and fabrication technologies specifically available in foot and ankle prosthetics, with a focus on AM methods.

## 2. Total Ankle Replacement

**Figure 2:**
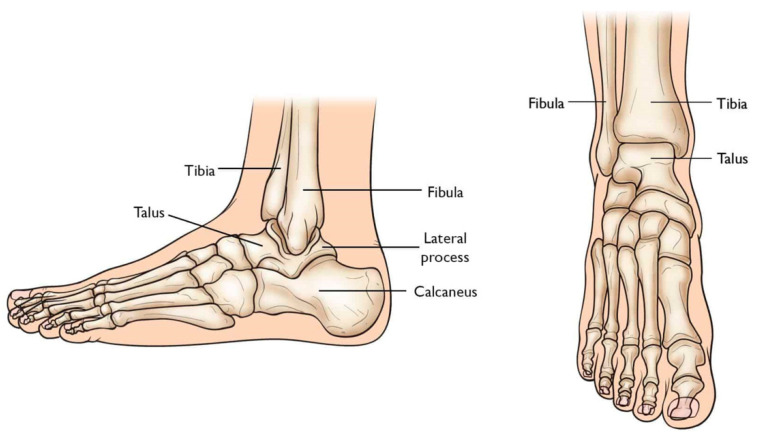
Graphic of bones in ankle joint, including talus, fibula, tibia, calcaneus, and surrounding bones [20].

Total Ankle Arthroplasty (TAA) is an orthopedic procedure used as an increasingly popular treatment for patients with end-stage arthritis in the ankle joint. The ankle joint, also known as the tibiotalar joint, is formed from the meeting of the tibia, fibula, and talus bone [18], shown and labeled in Figure 2. Wearing of the cartilage within the joint can lead to the bone ends rubbing against each other, causing bone spurs, inflammation, stiffness, osteophyte formation, and pain in the joint, overall resulting in severely decreased function [18,19]. 

### 2.1. The Evolution of Total Ankle Arthroplasty Implants

Previously, ankle arthrodesis (AA), which consists of fusing two or more bones within the joint together, was considered the standard treatment for end-stage ankle arthritis [21]. While studies have reported patient satisfaction following AA as very high, assessment of functional outcomes has shown reduced mobility of the joint during activities of daily living as well as post-surgical degeneration of the neighboring subtalar joint [21,22,23,24,25].

**Figure 3:**
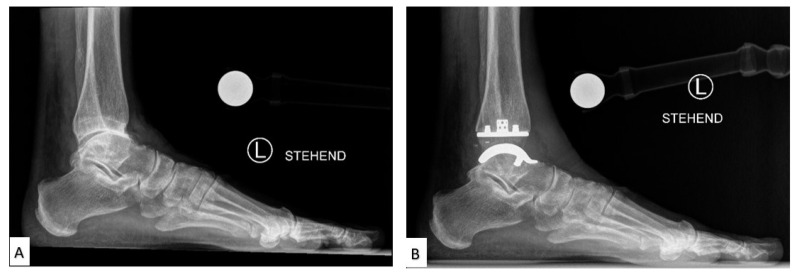
Labeled X-ray of (**A**) preoperative and (**B**) TAA implant performed on a patient, consisting of a polyethylene tibial component and metallic talar component [26].

TAA was developed as a method of reducing pain while also preserving the mobility of the joint. Early first-generation TAA implants (Figure 3) consisted of two components: a concave tibial component composed of polyethylene and a convex talar component composed of metal alloy, both fixated with bone cement. However, early TAA implants are still associated with a high rate of failure, which has been attributed to permanent deformation of the polyethylene component, large bone resection to allow for cement fixation and loosening of the talar component due to the high strength of the talus bone [21,23,24,25].

### 2.2. Current Total Ankle Arthroplasty Implants

**Figure 4:**
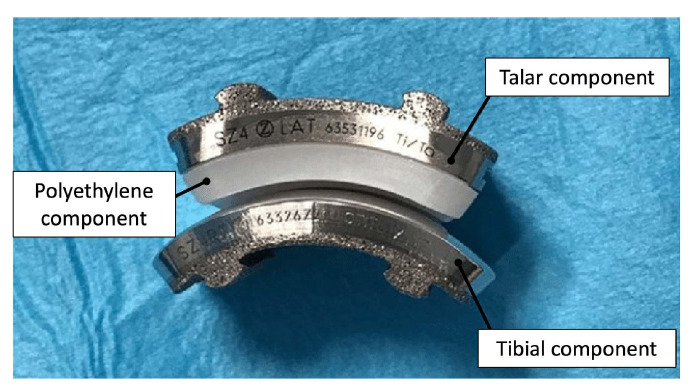
Photograph of 3-part TAA implant composed of a metal tibial component, a mobile polyethylene insert, and a metal talar component [30].

Second through fourth-generation TAA implants consist of three components: a metal component attached to the tibia, a metal component attached to the talus, and a mobile plastic implant between the two [27,28], as shown in Figure 4. Advancements in this design provided almost normal kinematic function of the joint in plantarflexion/dorsiflexion, inversion/eversion, and tibial rotation, resulting in overall improved gait performance as well as increased pain relief compared to AA outcomes [26,28]. Existing reviews have concluded that complication rates in patients with TAA (19.7%) in recent years have also been lower than in patients with AA (26.9%) [29]. However, complication and failure rates remain higher than those for knee and hip arthroplasties, still making TAA a controversial technique in clinical use. Revision rates following TAA have also been reported as high as 7.9%, compared to 5.4% for AA [29], which has been largely attributed to the loosening of the implant.

### 2.3. Advancements in Total Ankle Arthroplasty Design and Manufacturing

Loosening of TAA implants has been attributed to the mal-positioning of the implant, leading to altered motion and pressures at the contact surface [24]. Furthermore, end-stage osteoarthritis (OA) can lead to significant degradation of the articular surface of the tibia and talus, which may lead to deformity of the underlying osteochondral bone and properties of the supporting soft tissue. Thus, mass-produced implants limited to mimicking the anatomy of a healthy joint can be highly unstable and unsupported in an OA joint. However, AM permits the fabrication of personalized TAA implants and reduces the risk of implant failure and patient discomfort.

**Figure 5:**
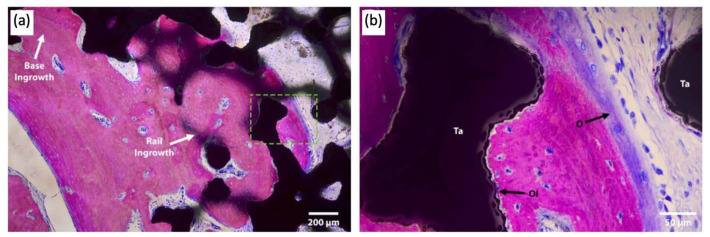
Light microscopy images of the talar/bone component stained with Sanderson’s rapid bone stain (SRBS) 3.5 years post-operation. Mineralized bone was stained pink, osteoid and non-mineralized soft tissue were stained blue, and the color black is for tantalum. (**a**) Low-magnification image of explants showing bony ingrowth into the porous tantalum implant, with healthy remodeling. (**b**) Higher-magnification view of the green dotted box in image (**a**) showing osseointegration to the porous tantalum with the patient’s newly formed bone. Ta: tantalum, O: osteoid, OI: osseointegration [32].

Attention has been paid to the bone–implant interface of TAA implants. In addition to hydroxyapatite and porous surface coating technologies commonly used in other joint replacements, a recent case study reported the use of a third-generation TAA implant, comprised of a titanium alloy tibial component and cobalt–chromium (CoCr) talar component, both coated with porous tantalum (P-Ta) (80% porosity) [31]. Three years following implantation, the authors performed a histological analysis of the intact implant (Figure 5). Results showed significantly increased bone ingrowth into the P-Ta layer of 9.4% to 13.6% (previously reported as 1–5%), allowing for increased initial bone stability and symmetric loading [32]. 

Given the instability of TAA implants in cases of malalignment, advancements have also been made in surgical planning technologies consisting of patient-specific CT scans and 3D-printed bone models. Studies have shown that planning using patient-specific bone models allows for increased reliability and reproducibility in the surgical placement of TAA implants [33,34].

**Figure 6:**
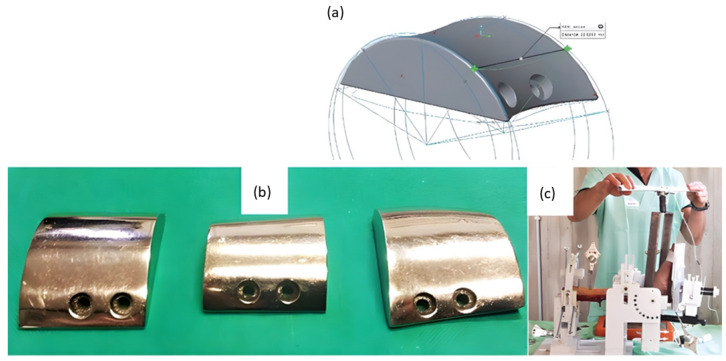
Fabrication of cobalt–chromium–molybdenum 3D implants using SLM technology for TAA replacement. (**a**) Computer model of talar component of cadaveric specimen and rendering of talar component with matching curvature to demonstrate the process of bone modeling and custom prosthesis design. (**b**) 3D-printed talar components polished with a mirror finish. (**c**) Mechanical testing of implanted custom 3D-printed implants using a cadaver specimen on a testing rig. Modified from [35].

A group recently outlined their use of AM and testing of patient-specific TAA implants [35]. CT scans of the tibia, fibula, talus, and calcaneus bones of anatomical cadaver specimens were used to design computer models of the implant with matched curvature radius and external contouring of the bones (Figure 6). Selective Laser Melting (SLM) using cobalt–chromium–molybdenum powder (spherical, 15–45 µm) was then used to create near-full density implants for the two metal components of the implant. In vitro testing of the components implanted on corresponding cadaveric specimens was performed by applying loading/unloading cycles in three dimensions. Joint mobility and stability were tested through manipulation in dorsiflexion/plantarflexion.

**Figure 7:**
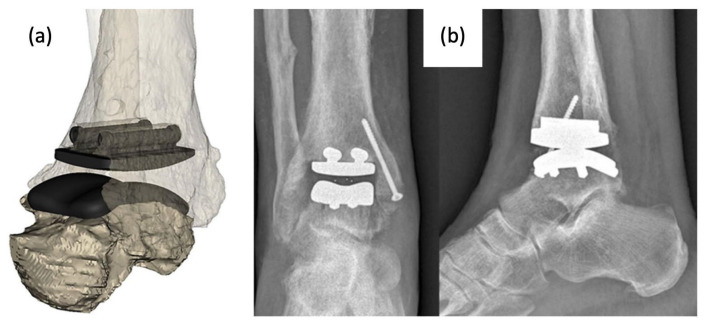
(**a**) Computer model of custom-designed implant on patient CT scan rendered bone model and (**b**) anterior–posterior and lateral X-rays of implanted AM TAA implant [36].

More recently, a group released a case report of the use of a patient-specific AM TAA implant in a patient when standard, modular TAA implants were found to be too small for the patient’s anatomy. A CT scan of the mid-tibia to the whole foot was obtained and analyzed to model the morphology of the joint as well as existing bone resections (Figure 7). The metal components were created using AM with cobalt–chromium–molybdenum. Following implantation, the patient was shown to regain activities of daily living and experience no pain after 4 months. Gait analysis at this time point also revealed a quasi-physiological pattern revealing limited deficits in the joint with normal muscle activation aside from prolonged activation in the gastrocnemius [36].

## 3. Total Talus Replacement

The talus articulates with the fibula and tibia to form the ankle joint, allowing dorsiflexion and plantarflexion of the foot, and with the calcaneus to make the subtalar joint, allowing inversion and eversion of the foot (for anatomical pictural representation, reference Figure 2: Graphic of bones in the ankle joint, including talus, fibula, tibia, calcaneus, and surrounding bones). It is responsible for the transmission of weight and pressure forces from the lower leg to the foot and is covered by articular cartilage for smooth movement against its neighboring bones [37].

Total talus arthroplasty (TTA) is an alternative orthopedic procedure that was developed as a treatment in cases of total talar compromise due to avascular necrosis, trauma, osteonecrosis, tumor-induced bone defects, or talar body collapse due to complications of TAA [18,38,39,40,41,42,43,44].

### 3.1. The Evolution of Total Talus Arthroplasty Implants

Prior to the advent of TTA, the recommended treatment for trauma or avascular necrosis of the talus was either arthrodesis or talectomy paired with simultaneous tibiocalcaneal arthrodesis; however, both options often resulted in the loss of ankle and foot function, as well as hindfoot instability in the former and discrepant leg shortening in the latter. TTA was designed to replace the talus while preserving the motion and work of the ankle and foot [38].

**Figure 8:**
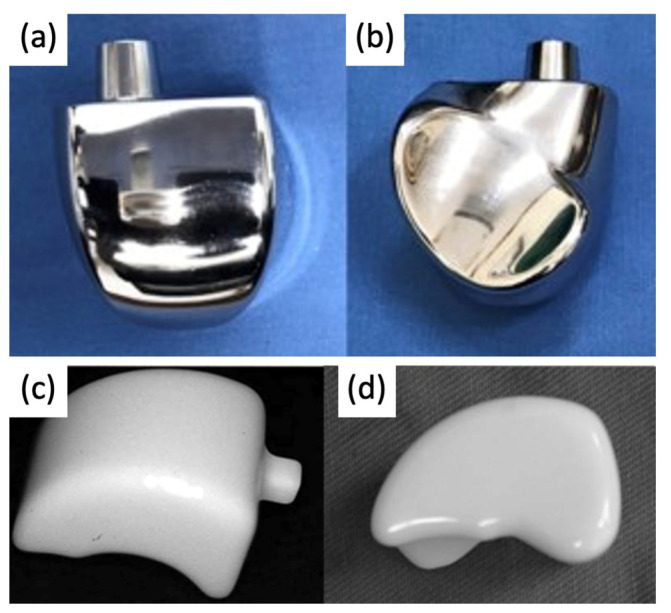
(**a**,**b**) First-generation talar body prosthesis with an attachment peg and (**c**,**d**) second-generation prosthesis [38,40].

The bone consists of three parts: head, neck, and body. In first-generation TTA implants (Figure 8), the prosthesis consisted of two components: the talar body and a peg for attachment into the talar neck. Only the talar body was replaced, and the talar neck was fixated on the prosthetic stem with bone cement. Satisfactory results were reported in post-operative evaluations, with preserved joint stability and increased mobility of the ankle and foot. However, there were noted issues with prosthesis congruence and prosthesis failure where the prosthesis stem had sunken into the talar neck [38,39].

In second-generation TTA implants (Figure 8), the peg was removed to address the previously reported sinkage and subvert the concentration of stress in the talar neck. The talus body was surgically placed without fixation. While radiological appearances were more satisfactory, second-generation implants could not be recommended as a treatment for avascular necrosis due to the high degree of loosening seen between the prosthesis and talar neck [40].

### 3.2. Current Total Talus Arthroplasty Implants

**Figure 9:**
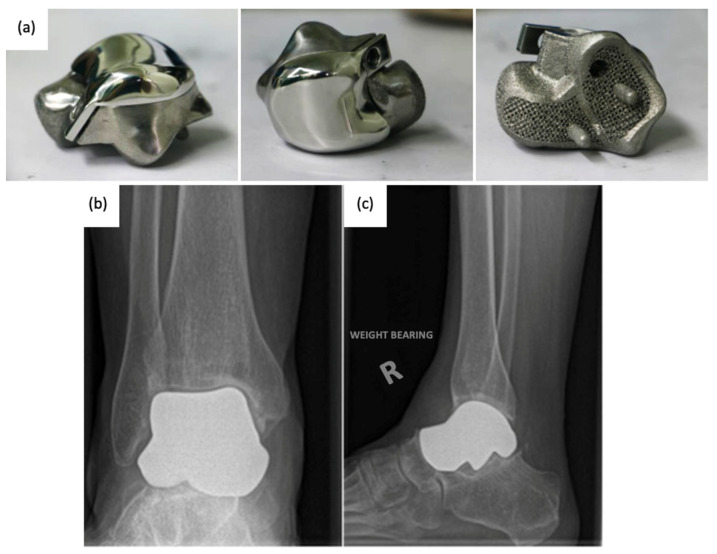
(**a**) 3D-printed third-generation total talus prosthesis and post-operative X-rays of implanted 3D-printed third-generation TTA prosthesis, (**b**) anterior–posterior, and (**c**) lateral views [46,49].

Since the talus is not stabilized by any muscular attachments, its positioning primarily depends upon the support of the surrounding bones and ligaments, and thus, the most important factor for prosthesis survival is implant congruence with articular surfaces [45]. Recently introduced third-generation TTA implants (Figure 9) are described as custom total talar prostheses, which circumvents the earlier concerns of loosening and sinking between the talar neck and prosthesis. Many groups have designed TTA implants using the patient’s healthy contralateral talus as a model for AM, which can be made into forms that may not have been possible to achieve using the current production method [46]. Custom third-generation TTA implants have been manufactured primarily by SLS, using materials including cobalt–chrome, nickel-plated cobalt, titanium alloy, and alumina ceramics. These implants have demonstrated retention of mobility, rapid pain relief, and preserved joint height, as well as a shorter duration of restricted weight bearing [42,43,44,47,48]. A case series evaluating the outcomes of patients who underwent TTA with AM titanium or cobalt–chromium talus implant reported no complications at a mean follow-up of 36 months with preserved range of motion in the ankle and improved pain scores [49]. To date, there have been no issues of prosthesis size incongruence, but long-term follow-up is needed to evaluate the longitudinal durability of custom-made TTA implants and their effects on ankle and foot function. Potential complications of concern with third-generation TTA may involve displacement of the implant.

### 3.3. Advancements in Total Talus Arthroplasty Design and Manufacturing

Third-generation TTA with complete, custom talus implants has shown promising results for post-operative pain relief and mobility; however, the current production method is costly and slow. In a 55-patient case study examining the use of ceramic TTA implants, CT imaging was used to generate a wire model, from which a stereolithographic model was generated. From the model, an alumina-ceramic prosthesis was created, with the entire manufacturing process taking approximately 4 weeks [43]. As AM becomes an increasingly popular method for TTA implant manufacturing, attention is being paid to faster prosthesis design, resulting in optimized anatomical fit within a shorter timeline.

Furthermore, the use of metal Additive Manufacturing allows for a more flexible prosthesis design, where a broader range of metals can be utilized to devise an optimal fit. With topology optimization, unnecessary weight from materials within the inner spaces of the implant can be eliminated for a lightweight design that improves comfort [46,50]. To address the development of osteosclerosis due to incongruent elastic modulus between prosthesis materials and bone, the use of ultra-high-molecular-weight polyethylene (UHMWP) on the articulation of the prosthesis with the tibia has been promoted as a potential substitute for wear reduction [51].

## 4. Ankle Prosthetics

Ankle prosthetic devices are indicated for patients with various lower extremity impairments, including lower limb amputations resulting from trauma or underlying disease, as well as neurological deficits due to spinal cord injuries or strokes. In patients with lower extremity amputations, recent developments in ankle prosthetic devices aim to decrease metabolic costs and improve mobility and gait while simultaneously increasing comfort for the patient. Additionally, in patients with lower extremity neurological deficits, ankle prosthetics have the potential to alleviate many negative health consequences, including obesity syndrome, various chronic diseases, decreased bone health, and pressure injuries [52].

Ankle prosthetics can be divided into three main categories, including passive, quasi-passive, and active prosthetic devices, with studies providing evidence for the superiority of quasi-passive and active devices [53]. Passive prosthetics do not contain an external energy source, while active prosthetic devices, otherwise known as powered exoskeletons, have demonstrated significant improvements in functionality, including improved accommodation for terrain and faster walking speed [54]. Different prosthetic types are indicated based on varying lifestyles and activity levels [55].

### 4.1. Evolution of Ankle Prosthetics

**Figure 10:**
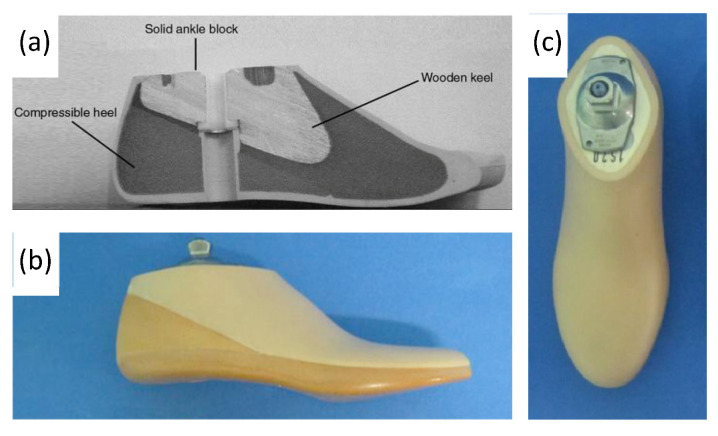
(**a**) Labeled drawing of a SACH foot cross-section, including wood, rubber, and compressible foam materials, and (**b**,**c**) photograph of SACH foot prosthetic including a cosmetic shell [59,60].

Conventional, passive prosthetic feet include the Solid Ankle Cushion Heel (SACH) foot and Energy Storage and Return (ESAR) prosthetics. After its introduction in 1980, the SACH foot became the standard due to its availability, inexpensiveness, and durability. The SACH foot is a basic design consisting of wood, rubber, and compressible foam materials (Figure 10). The SACH foot design has been shown to provide flexibility based on the height and weight of the patient and provide some degree of mobility in dorsiflexion only [56]. However, it is not appropriate for active amputees due to its lack of functionality compared with other ankle–feet prosthetics. Passive ankle prosthetic devices, such as the SACH foot, have been shown to produce less, approximately one-eighth of the power of intact gastrocnemius and soleus muscles [57]. It is now more commonly used as a temporary prosthetic device while patients decide on a long-term prosthetic option [58].

**Figure 11:**
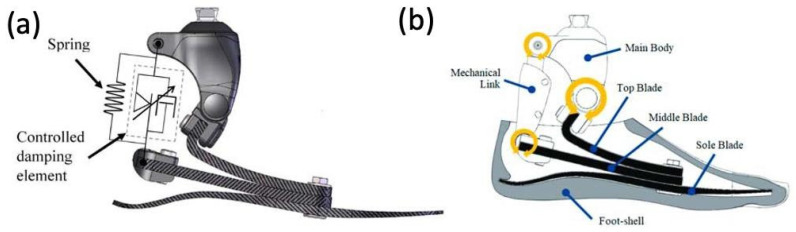
(**a**) Appearance of constructed functional ESAR prosthetic and (**b**) labeled graphic of the ESAR foot design with the top blade, middle blade, sole blade, mechanical link, and main body [62].

ESAR feet are designed to store elastic energy during midstance and subsequently release energy during push-off. Multiple variations of the ESAR foot exist, with the basic design consisting of multiple flexible blades attached to an adaptor with a mechanical link (Figure 11). ESAR implants are typically composed of carbon fiber or carbon fiber-reinforced polymers, and groups have also employed AM using SLS to manufacture patient-specific implants with desired stiffnesses to improve gait [61].

**Figure 12:**
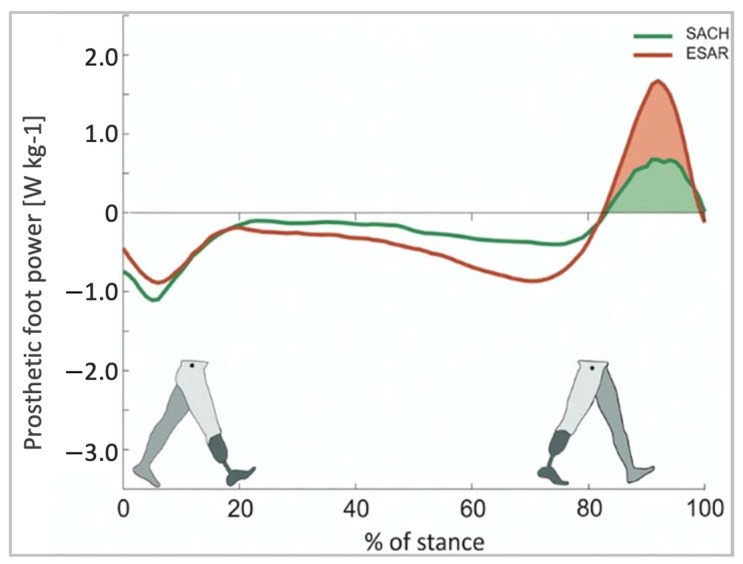
Push-off power of the prosthetic foot as a function of normalized stance time [64].

ESAR prosthetic feet generate greater energy, reduce metabolic costs, and increase self-selected walking speeds when compared to the conventional SACH foot [63]. A study comparing the ESAR foot to SACH feet demonstrated that the ESAR foot allowed for higher push-off power and increased intact step length without decreasing backward stability [64] (Figure 12). 

**Figure 13:**
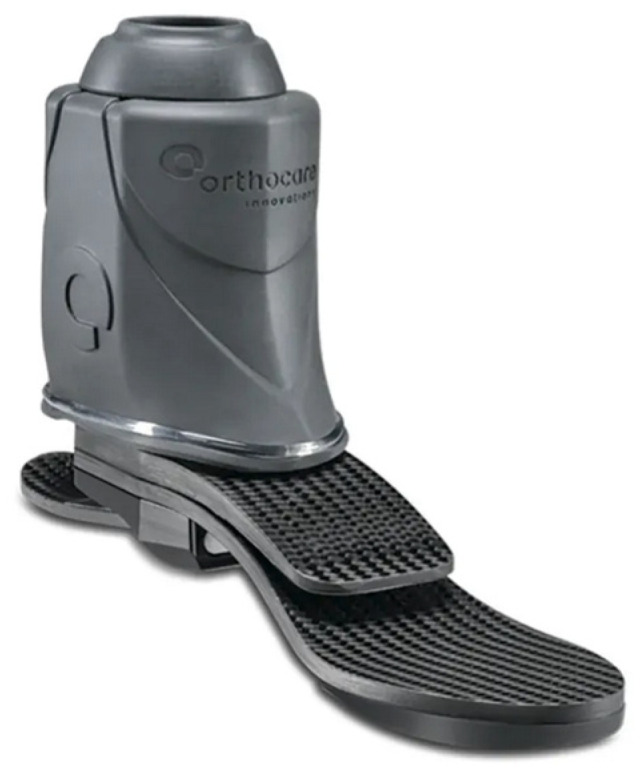
A commercially available modern MPF advertised as an electronically controlled ankle joint that allows adjustment to user’s walking speed and ground conditions [68].

Recent developments in powered ankle exoskeletons (PAEs) aim to reduce the energy expenditure of patients with lower extremity amputations and improve their quality of life [65]. PAEs utilize microprocessors, which are internal computers with sensors that relay information between the human body and the device. Microprocessor-controlled ankle–foot (MPF) systems were commercially introduced in 2006 with the goal of improving gait mechanics. The original MPF system, however, lacked the ability for powered plantarflexion. In more recent years, MPF prosthetic devices have been developed to more closely mimic physiological ankle function (Figure 13) [66,67]. MPFs have shown multiple advantages compared to non-microprocessor prosthetic feet, including improvement in biomechanical performance during climb of ramps and stairs, increased toe clearance during swing, reduced pressures against remaining limb within the socket, and increased mobility of the prosthetic [66]. Additionally, significant improvements in physical function scores were found in prosthesis users who transitioned from using non-MPFs to MPFs [66]. Common materials used in MPF ankle systems include carbon fiber and titanium.

Microprocessors for powered exoskeletons can be divided into two main categories: those that utilize neural control versus those that utilize mechanically intrinsic control [69]. Neural control relies on brain or muscle electrical activity, interpreting these signals and coordinating actions of the device appropriately. In mechanical control, the device combines information on gait events, joint angles, and forces to predict human intention. A direct comparison study found that plantar flexor muscle recruitment was less in participants using time-based controllers compared to myoelectric control [69]. No metabolic work rate differences were noted between myoelectric-controlled prosthetics compared to mechanically intrinsic control. Further research is needed to determine the superiority of neural versus mechanical control.

### 4.2. Early Powered Exoskeletons in Gait Rehabilitation

**Figure 14:**
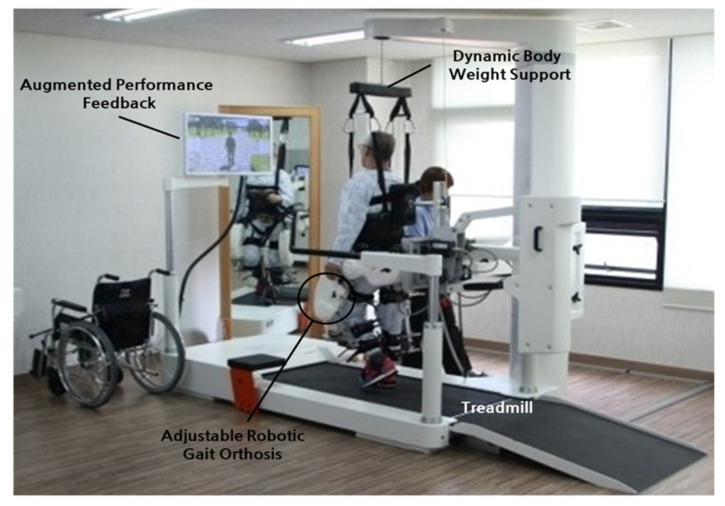
Lokomat device used for gait rehabilitation following lower-limb disability, brain injury, or spinal cord injury. The device consists of a treadmill, body weight support, and powered leg orthosis [70].

Early robot-assisted gait training was first initiated in a clinical therapeutic setting, often attached to training devices such as treadmills. These initial models were designed as orthotic devices with body-weight support. For instance, the Lokomat (Figure 14) is composed of a motorized exoskeleton that provides guidance forces onto the knee and hip joints and has been shown to increase the range of motion about the ankle joint. Briefly, the motorized exoskeleton consists of a DC motor with helical gears to direct the trajectory of the joints [70]. The Lokomat device has been used successfully in gait rehabilitation for patients with brain or spinal cord injuries that exhibit muscle weakness, spasticity, or abnormal muscular activation patterns. Previous forms of therapy used body weight support with assistance from physical therapists, but recent advancements have allowed for motorized exoskeletons to provide these additional forces. This allows therapy sessions for patients to increase in both intensity and duration while also maintaining physiological gait patterns.

Varoqui et al. utilized a 4-week Lokomat training program on patients with incomplete spinal cord injuries due to trauma and impaired voluntary ankle movements [71]. Their outcomes showed significant improvement in the range of motion of the ankle. Kinetic and kinematic measurements also demonstrated voluntary movements of the ankle were farther and faster after training. Additionally, patients demonstrated an increase in dorsiflexion strength, which is significant as patients with spinal cord injuries often have gait issues from foot drop syndrome.

### 4.3. Battery-Powered Exoskeletons in Congenital Disorder Gait Rehabilitation

As powered exoskeletons advance to more mobile forms that allow patients to move overground in an unrestricted area, applications are being explored in congenital neurological disorders like cerebral palsy (CP). Previous therapies for these patients with pathological gaits that ultimately progressed to reduced walking ability in adolescence included physical therapy, orthotics, muscle injections, and surgeries, which did not necessarily show improvement in gait. The possibility of using wearable battery-powered exoskeletons is being explored to reinforce more favorable walking patterns in CP patients [72].

**Figure 15:**
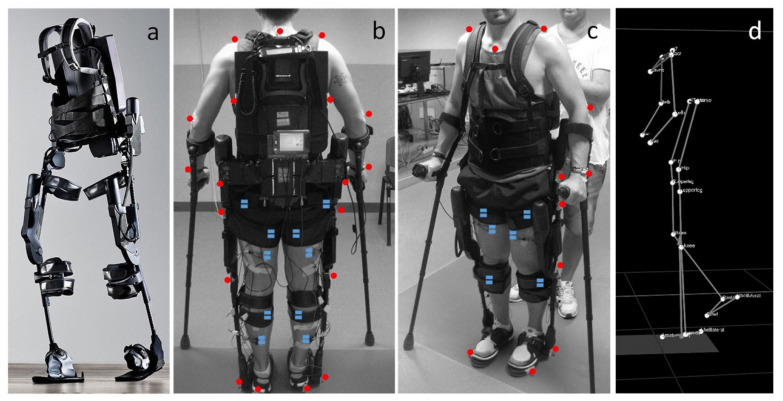
(**a**) Photograph of exoskeleton Ekso 1 equiped with 4 powered motors at the hips and knees, 2 powered joints at the hip and knee, and a semi-rigid unpowered ankle joint; (**b**,**c**) patient utilizing mobile ankle exoskeleton with the aid of crutches to maintain balancing. The system was equiped with motion analysis markers (red dots) and electromyographs (blue dots) for gait analyis; (**d**) kinematic data resulting from the motion capture system [72].

These exoskeletons (Figure 15) are composed of an onboard battery that powers high-performance DC motors. This structure is actuated onto a pulley aligned with each ankle joint via a Bowden cable transmission that helps to mount the heaviest components of the exoskeleton onto the patient’s torso. In addition, torque sensors are mounted in line with the exoskeleton’s ankle joint, allowing for feedback-based motor control. Under the ball of each foot, additional foot sensors allow for the implementation of the plantar flexion assist on the mode of the exoskeleton, adding torque to the ankle joint to mimic the shape and timing of normal physiological ankle movement.

Patients of varying ages and severity of gait pathologies due to CP disease progression showed improved lower extremity posture, increased propulsive ankle joint point, and reduced plantar flexion activity, which was shown to be a contributing factor toward gait abnormalities. Augmentation with the powered exoskeleton also showed more efficient walking patterns, meaning the additional ankle assistance provided by the exoskeleton was reducing the use of additional muscles at the hip joint for walking and, therefore, improved contractures seen in these patients. Overall, the wearable exoskeleton was shown to be well-tolerated by patients and has the potential for long-term gait improvements in CP patients.

### 4.4. Powered Exoskeletons in Stroke Rehabilitation

Stroke patients commonly suffer from foot drop syndrome, which equates to a higher falling risk, making effective gait rehabilitation essential to improve patients’ quality of life and independence. Prior gait training systems generally confined patients to a treadmill-like setting and may not contribute to the ultimate goal of independent walking on real-world surfaces. Passive ankle–foot orthotics have also been used in conjunction with conventional physical therapy, but they also limit the patient’s range of motion about the ankle joint.

**Figure 16:**
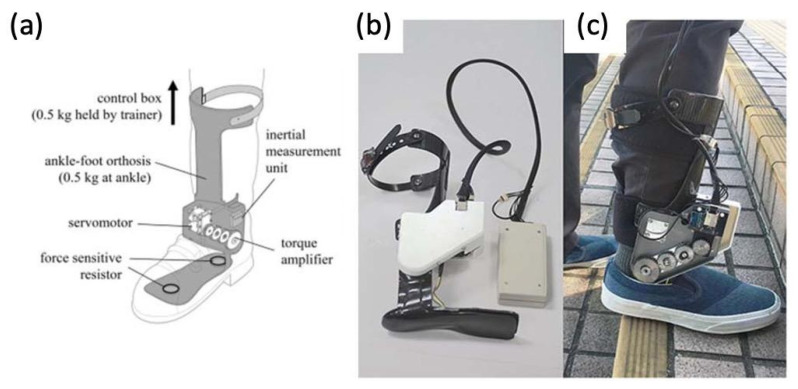
(**a**) Graphic of powered ankle exoskeleton used in gait training of stroke survivors, including labels of rotatory servomotor and torque amplifier and (**b**,**c**) photographs of a manufactured powered ankle exoskeleton used on stroke patients [73,74].

Powered exoskeletons are being applied in the gait rehabilitation of both chronic and sub-acute stroke patients [73,74]. One such model being used is a modified form of an ankle–foot orthotic coupled to a rotatory servomotor and torque amplifier that provides powered assistance in plantarflexion and dorsiflexion (Figure 16). The powered exoskeleton also contained force-sensitive resistors that could identify changes in gait phase and foot loading.

After multiple sessions of gait rehabilitation, including walking over-ground and stair training, stroke patients were able to walk with greater speed and higher cadence compared to those just undergoing conventional physical therapy. This finding was also true once robotic assistance was removed in clinical assessments, suggesting further implications for gait-relearning applications in the future. It was also shown that patients had improved gait patterns, more independence with walking, and increased walking speed, which has been associated with lower fall risk.

### 4.5. Powered Exoskeletons in Lower Extremity Trauma Patients

**Figure 17:**
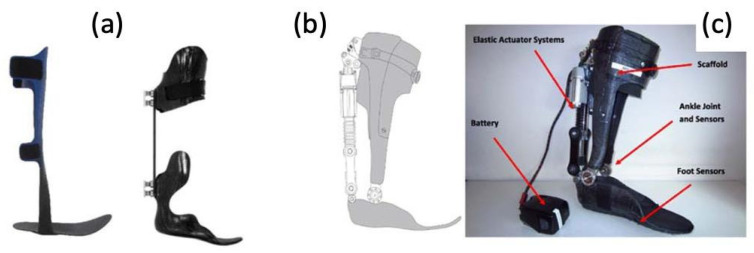
(**a**) Photographs of traditional, passive AFOs and (**b**,**c**) a graphic and photograph of a powered PFO, including labels of elastic actuator and ankle/foot sensors [75].

More and more are lower extremity trauma patients opting for limb reconstruction surgeries over amputations, making them candidates for wearable foot and ankle exoskeletons, termed ankle–foot orthotics (AFOs). More traditional forms of AFOs are passive (Figure 17). Although they can function to improve walking abilities in lower limb muscle weakness, they cannot fully restore plantarflexion push-off, still limiting gait as the ankle can only return to a neutral position instead of producing more peak power as in an intact ankle. The introduction of powered AFOs provides assistive torque that their passive counterparts are not able to provide. This allows for greater ankle range of motion, active dampening when the heel strikes the ground, and powered push-off during plantarflexion.

In a study involving three male Service Members who had sustained lower extremity injuries requiring surgical interventions [75], the use of their current passive AFOs was compared with the PowerFoot Orthosis (PFO), a powered advanced AFO. The PFO, which was powered by a lithium polymer battery, was formed by a carbon fiber scaffold structure that was customized to each Service Member. It contains a series-elastic actuator that generates net positive work about the ankle joint. This provides assistance by increasing dorsiflexion to allow for toe clearance and preparation for foot strike and for powered plantarflexion during push-off. Angular sensors, spring systems, and state controllers are used to vary torque and joint position in response to each Service Member’s step-to-step variations and walking phase.

Overall, the range of motion of the ankle joint and peak power generation at push-off was greater in the PFO compared to each Service Member’s previous passive AFO. It was found that the PFO has the potential to restore a more biomimetic gait by providing power torque with plantar flexion and assisting with dorsiflexion during swing. When asked about preference among the devices, all three Service Members still preferred their previous passive AFO. Their major concern was difficulty or discomfort with lateral movements and walking backward. However, their comments did reflect that they may have preferred the PFO had the construct been less bulky and lighter in weight.

## 5. Conclusions and Future Perspectives

In this review, we focused on a variety of implants used for foot and ankle prostheses and the evolution of material/fabrication processes. Broadly, trends in the field are moving towards personalized prostheses to overcome limitations related to patient discomfort or implant failure attributed to traditional designs. Furthermore, the potential of AM to manufacture prostheses with complex geometries at a high speed has attracted the interest of the scientific community. AM is currently being employed to fabricate implant components using materials, including ceramics, metals, or plastics. AM is also being used to manufacture customized sensors and actuators that can be assembled in a manner to improve patient mobility and rehabilitation. Attention is now being turned to combining advances in AM, imaging technologies, and robot-assisted surgery to manufacture custom-made guides for osteotomies that not only take into consideration the patient’s anatomy but also will allow a perfect fit between the native tissues and the prosthesis.

However, there are still challenges in substituting conventional technologies for AM technologies. Among them is the cost of the 3D printers, manufacturing, and assembly of the prosthesis, calling into consideration factors such as in-house printing versus local or global fabrication and shipping. At the current stage, many implants are developed by individual academic or industry groups using different AM technologies, materials, electronics, and post-treatment processes, making a cost analysis study between AM and traditionally manufactured implants hard to perform. Furthermore, despite the outstanding advancements in metals and polymers used in AM, more specific and oriented research needs to be conducted and applied to the prosthesis manufacturing field, including the development of multi-material printers and automation of multiple 3D printing technologies and processes. For example, stereolithography (SLA), digital light processing (DLP), and liquid-crystal display (LCD) printers use resin-based materials that need extensive washings and curing. Metal and ceramics printing involves extra steps for debinding and sintering, and sometimes, a polishing or plasma spray treatment is needed.

One step further than the personalization of prosthetic devices is the development of personalized exoskeletons in combination with human-in-the-loop optimization. Traditionally, collecting data on individual performance can be a time-consuming and expensive process. However, recent progress in developing wearable sensors and rapid methods of analysis [76] can measure and analyze the patient as well as the prosthetic performance data in real time. With the aid of artificial intelligence, the system energy storage and motor torque continuously change, overall enhancing performance with the ultimate goal of improving the patient’s quality of life and satisfaction. As AM technology continues to evolve, it plays an increasingly significant role in medicine. In addition to the direct manufacturing of foot and ankle implants and devices, AM patient-specific models can aid in surgical planning in complex cases and visualization of anatomy in medical education, further contributing to bettering the quality of patient care [77,78]. The AM of personalized implants used for ankle and foot prostheses and orthosis is an advanced interdisciplinary research area requiring cross-talk and collaborations from multiple research fields.

## Data Availability

Not applicable.
